# Spatial Distribution, Contamination Levels, and Health Risk Assessment of Potentially Toxic Elements in Household Dust in Cairo City, Egypt

**DOI:** 10.3390/toxics10080466

**Published:** 2022-08-11

**Authors:** Ahmed Gad, Ahmed Saleh, Hassan I. Farhat, Yehia H. Dawood, Sahar M. Abd El Bakey

**Affiliations:** 1 Geology Department, Faculty of Science, Ain Shams University, Cairo 11566, Egypt; 2 National Research Institute of Astronomy and Geophysics (NRIAG), Cairo 11421, Egypt; 3 Geology Department, Faculty of Science, Suez University, El Salam City 43518, Egypt; 4 Department of Biological and Geological Sciences, Faculty of Education, Ain Shams University, Cairo 11341, Egypt

**Keywords:** potentially toxic elements, indoor dust, pollution, exposure, risk assessment, urban, Cairo

## Abstract

Urban areas’ pollution, which is owing to rapid urbanization and industrialization, is one of the most critical issues in densely populated cities such as Cairo. The concentrations and the spatial distribution of fourteen potentially toxic elements (PTEs) in household dust were investigated in Cairo City, Egypt. PTE exposure and human health risk were assessed using the USEPA’s exposure model and guidelines. The levels of As, Cd, Cr, Cu, Hg, Mo, Ni, Pb, and Zn surpassed the background values. Contamination factor index revealed that contamination levels are in the sequence Cd > Hg > Zn > Pb > Cu > As > Mo > Ni > Cr > Co > V > Mn > Fe > Al. The degree of contamination ranges from considerably to very high pollution. Elevated PTE concentrations in Cairo’s household dust may be due to heavy traffic emissions and industrial activities. The calculated noncarcinogenic risk for adults falls within the safe limit, while those for children exceed that limit in some sites. Cairo residents are at cancer risk owing to prolonged exposure to the indoor dust in their homes. A quick and targeted plan must be implemented to mitigate these risks.

## 1. Introduction

Over the past few decades, a tremendous amount of hazardous waste materials has been released into various environmental media at increasing levels because of the rapid urbanization and globalization of economic and industrial activity [1,2,3,4,5]. Because the air in common is the primary carrier of fine particles, air pollution has produced a significant environmental impact (e.g., climate change and human health). The concentration of suspended particles in the air, which transports contaminants, especially potentially toxic elements (PTEs), has progressively increased, endangering humans. Because of their genotoxicity, carcinogenicity, chemical persistence, and non-degradability, PTEs attached to suspended particles would enrich in surface environments and have an acute or chronic impact on the health of vulnerable residents once they get into the human body [6,7,8,9,10]. PTEs can go through a human body via respiratory inhalation, ingestion of contaminated media, and dermal contact and accumulate over time [1,6,11,12].

Because indoor air can be significantly more polluted than outdoor air, it has captures remarkable attention from researchers. Imperfect air exchange and specific indoor emission sources combined with outdoor sources seems to be the leading causes of indoor air being a complex and contaminated environment [1,12]. People in megacities typically spend 80–90% of their own time indoors, in private homes, schools, and offices, potentially increasing their exposure to toxic substances being emitted from construction materials, household equipment, and electronic products, in conjunction with anthropogenic sources [13]. In this regard, the indoor ambiance and potential health risks inextricably associated with toxic substances’ exposure in the indoor environment must be considered. Many scientific studies over the last decades have sufficiently demonstrated that prolonged exposure to contaminated indoor environments has undeniable fingerprints on serious health problems that result from direct and indirect exposure [6,7,12,13]. This direct impact on public health is extremely significant for children, who are more vulnerable to contaminant exposure due to increased hand-to-mouth interactions [1,4,14]. Furthermore, considerable advancements in analytical techniques used to investigate various biological samples will progressively improve exposure estimates for both healthy and at-risk populations [15,16].

There are numerous sources of indoor contaminants, the most significant of which is settled and suspended dust. Most of these hazardous and toxic pollutants are adsorbed to suspended particulates in indoor air and later deposited as house dust. Because of this process, the concentrations of contaminants in indoor dust are higher than their natural crustal concentrations [6,12]. Indoor dust is a motley mixture of inorganic and organic materials that can adsorb and concentrate PTEs [17,18]. This admixture would settle on the surfaces of residential objects (e.g., floors, carpets, furniture, and others) [19,20]. The main pathway for PTEs from outdoor sources into homes is the entry of contaminated suspended particles into outdoor air [20,21]. Many transporting methods bring street dust and soil materials indoors as a consequence of residents’ activities (e.g., attached to shoes, clothes, bags, their pets, etc.) [7,12,22,23,24]. Moreover, considered external contaminated sources of indoor and household dust are suspended grains generated by industrial activities, road dust, traffic emissions, park soil, and other particles that are produced by outdoor activities [6,12]. Indoor dust PTE contamination has received a lot of attention owing to its significant effects on both residents’ health and the environment [7,13,25]. One of the serious issues with indoor dust is that it is not exposed to the same processes that reduce its PTE concentrations as those that affect outdoor dust (e.g., diluting, leaching, or weathering). Consequently, indoor dust could be used as a long-term indicator of indoor environmental status [19].

Egypt has experienced severe soil, water, and air pollution in recent decades as deleterious consequences of rapid economic growth, urbanization, and increased energy demands [26,27,28,29]. Different studies were conducted to assess Egypt’s air pollution. The vast majority of these studies have concentrated on the gaseous (CO, CO_2_, SO_2_, H_2_ S, and NO_2_) and particulate matter [30,31,32,33,34,35]. Studies on indoor dust in Egypt have typically focused on major ions (SO4, NO_3_, Cl, NH_4_, Ca, Mg, Na, and K) [36], organic pollutants [37,38,39,40], and microorganisms [41,42,43]. There are limited studies on PTE contamination in indoor dust and their health risk assessments [23,24,44,45]. Moreover, no comprehensive geochemical study of Cairo City’s indoor dust and the potential health risks for PTE exposure have been conducted. A notable lack of such necessary data might hinder the proper development of short and long-term policy initiatives towards reducing air pollution. More extensive research must be directed to thoroughly comprehend the detrimental health impacts of PTE air pollution. Findings and the conclusion of these surveys will be properly utilized to support the national policies and will contribute to the public health improvements. Therefore, the current study’s specific objectives are to (1) detect the PTE levels in household dust and identify their spatial distribution in Cairo City; (2) assess the contamination levels using environmental indices; (3) identify the possible sources of PTEs in household dust using multivariate statistical analysis; and (4) assess the potential health risk for children and adults’ exposure to PTEs.

## 2. Materials and Methods

### 2.1. The Study Area

**Figure 1:**
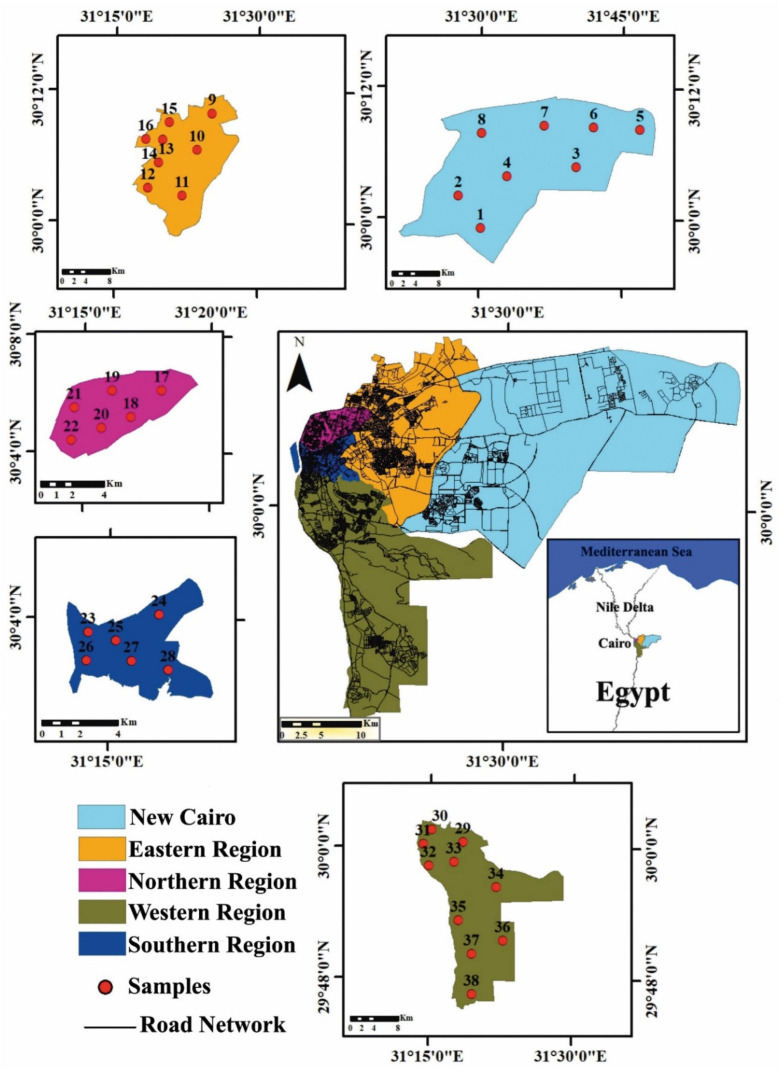
Map displaying Cairo City and its administrative regions and sampling site’s locations.

Cairo (Al-Qhirah) is located in northern Egypt on the River Nile’s right bank. It is Egypt’s administrative center and the most sizable city in both Africa and the Middle East, and one of the world’s most densely populated cities (9.9 million inhabitants). Many issues plague the city, including traffic congestion, air, soil, and water pollution, and ineffective waste management [46]. Cairo is administratively divided into five chief regions (New Cairo, Eastern, Northern, Western, and Southern) (Figure 1; Table S1).

Cairo City has a typical Mediterranean climate, with different temperatures through seasons: winter 14 °C, spring 21 °C, summer 36 °C, and fall 23 °C. Most of the year, wind speeds range from 3 to 8 m/s. The north and northeast were dominant wind directions [47,48]. It is surrounded by agricultural and industrial activities. It contains the main industrial zones that exist in the Northern and Southern regions which host cement manufacturing plants, steel, oil and gas, quarrying, rubber, petrochemicals, metallurgical, textile, and plastic products [26,48].

### 2.2. Sampling and Samples Preparation

A total of 38 composite household settled dust samples were collected from different regions and districts in Cairo City in 2021 (Figure 1). The sample size was selected based on the major districts in Cairo City, in conjunction with budgetary constraints. To ensure a collection of representative samples at least 10 subsamples were collected from each main district representing a total of 473 private houses (1 sample per house) (Table S1 in Supplementary Materials). The undisturbed surfaces, such as cupboards, fans, bookshelves, and refrigerators, were slowly brushed using precleaned polyethylene brushes and plastic dustpans to collect dust samples, which were then carefully blended and placed into transparent, zip-locked, and labeled plastic bags. The collected dust subsamples were carefully mixed and homogenized into 38 composite samples. The samples were then dried at 50 °C for 24 h in an oven followed by sieve analysis using a standard stainless-steel sieve (63 microns).

### 2.3. Chemical Analyses

The chemical analyses were performed using the ICP-ES/MS (AQ200) technique in ACME Lab, Vancouver, Canada (ISO 17025 and ISO/IEC 17025). An exact amount of 0.5 g of each household dust sample was leached in modified aqua regia (1: 1: 1 HNO_3_: HCl: H_2_O) [49]. Detection limits of Al, As, Cd, Co, Cr, Cu, Fe, Hg, Mn, Mo, Ni, Pb, V, and Zn were 0.01%, 0.5 ppm, 0.1 ppm, 0.1 ppm, 1 ppm, 0.1 ppm, 0.01%, 0.01 ppm, 1 ppm, 0.1 ppm, 0.1 ppm, 0.1 ppm, 1 ppm, and 1 ppm, respectively.

### 2.4. Contamination Levels

#### 2.4.1. Contamination Factor (*C_f_*)

Anthropogenic activities’ contribution to PTE contamination has been evaluated using the contamination factor (*C_f_*). *C_f_* is calculated by the Equation (1) [50].

(1) $$C_{f}^{i}=\frac{C_{s}^{i}}{C_{b}^{i}}$$

where $C_{s}^{i}$ is the PTE concentration in analyzed samples, and $C_{b}^{i}$ is the background value of the investigated PTE. In this investigation the Upper Continental Crust (UCC) element concentrations [51] were considered as the background values. The *C_f_* values are typically categorized in four distinct classes; class 1 (*C_f_* < 1.0 = low contamination); class 2 (1.0 ≤ *C_f_* < 3.0 = moderate contamination); class 3 (3.0 ≤ *C_f_* ≤ 6.0 = considerable contamination); and class 4 (*C_f_* > 6.0 = very high contamination) [50].

#### 2.4.2. Contamination Degree (*C_deg_*)

To detect multielement contamination, *C_deg_* was used. It was calculated for each sampling site using Equation (2) [50].

(2) $$C_{deg}=\sum_{i=1}^{n}C_{f}$$

where *C_f_* is contamination factor, and *n* is the number of the examined PTEs. The *C_deg_* values are typically categorized in four distinct classes; class 1 (*C_deg_* < 6.0 = low contamination); class 2 (6.0 ≤ *C_deg_* < 12.0 = moderate contamination); class 3 (12.0 ≤ *C_deg_* ≤ 24.0 = considerable contamination); and class 4 (*C_deg_* > 24.0 = very high contamination) [50].

### 2.5. Health Risk Assessment

PTEs measured in household dust in this investigation are typically known to possess noncarcinogenic effects on human health [52,53,54]. As, Cd, Cr, Ni, and Pb are believed to possess both noncarcinogenic and carcinogenic effects [52,53,54]. In the current study, health risks for children and adults in Cairo City were assessed using the noncarcinogenic Hazard Quotient (HQ) of a single element and Hazard Index (HI) of multiple elements via ingestion, inhalation, and dermal routes of exposure. Furthermore, the Cancer Risk (CR) was calculated using the concentrations of As, Cd, Cr, Ni, and Pb in the collected household dust samples. HQ, HI, and CR were calculated using the calculation model of exposure adopted by USEPA [52,53,54,55].

The average daily intakes (ADI) of PTEs in the household dust via nondietary inadvertent ingestion (noncarcinogenic) (*ADI_ing_*), dust inhalation (noncarcinogenic) (*ADI_inh_*), and dermal contact (noncarcinogenic) (*ADI_der_*) routes are calculated using Equations (3)–(5) as follows:

(3) $$ADI_{ing}=\frac{C_{s}\times IngR\times EF\times ED\times CF}{BW\times AT}$$

(4) $$ADI_{inh}=\frac{C_{s}\times InhR\times EF\times ED}{PEF\times BW\times AT}$$

(5) $$ADI_{der}=\frac{C_{s}\times SA\times SL\times ABS\times EF\times ED\times CF}{BW\times AT}$$

The noncarcinogenic risk *HQ* and *HI* of PTEs in the household dust is calculated using Equations (6)–(9) as follows:

(6) $$HQ_{ing}=\frac{ADI_{ing}}{RfD_{ing}}$$

(7) $$HQ_{inh}=\frac{ADI_{inh}}{RfD_{inh}}$$

(8) $$HQ_{der}=\frac{ADI_{der}}{RfD_{der}}$$

(9) $$HI=\sum^{\;}HQ_{ing}+\sum^{\;}HQ_{inh}+\sum^{\;}HQ_{der}$$

The lifetime average daily dose (carcinogenic) (LADD) and the carcinogenic risk (CR) of As, Cd, Cr, Ni, and Pb in household dust is calculated using Equations (10)–(13) as follows:

(10) $$LADD_{ing}=\left(\frac{C_{s}\times EF\times CF}{AT}\right)\times \left({\left(\frac{IngR\times ED}{BW}\right)}_{Child}+{\left(\frac{IngR\times ED}{BW}\right)}_{Adult}\right)$$

(11) $$LADD_{inh}=\left(\frac{C_{s}\times EF}{AT\times PET}\right)\times \left({\left(\frac{InhR\times ED}{BW}\right)}_{Child}+{\left(\frac{InhR\times ED}{BW}\right)}_{Adult}\right)$$

(12) $$LADD_{der}=\left(\frac{C_{s}\times \mathrm{SL}\;\times \mathrm{ABS}\;\times EF\times CF}{AT}\right)\times \left({\left(\frac{SA\times ED}{BW}\right)}_{Child}+{\left(\frac{SA\times ED}{BW}\right)}_{Adult}\right)$$

(13) $$R=\left(\sum^{\;}LADD_{ing}\times SLF_{ing}\right)+\left(\sum^{\;}LADD_{inh}\times SLF_{inh}\right)+\left(\sum^{\;}LADD_{der}\times SLF_{der}\right)$$

where all the abbreviations, definitions, and reference values are given and explained in Table 1. If *HI* is less than one, there is no risk of noncarcinogenic effect; if *HI* is greater than one, there is a risk of noncarcinogenic effect. A value of CR less than 1 × 10^−6^ is regarded as modest, a value of CR between 1 × 10^−4^ and 1 × 10^−6^ is regarded within the permissible level, and a value of CR greater than 1 × 10^−4^ is likely to be harmful to humans [52,53,54,55].

### 2.6. Data Treatment

Arc GIS (version 10.8.1; 2020) with a raster interpolation technique (Spline-Tension) was used to display the measured PTEs’ location and spatial distribution maps in Cairo City. OriginLab (version OriginPro 2021) was used to present descriptive statistics, boxplot figures, and multivariate statistical analyses. Excel (version Microsoft Office 365 16.0.15028.20160) was used to calculate contamination levels and health risk assessment.

## 3. Results and Discussion

### 3.1. PTE Distribution

This is the first investigation to present a multielement profile of Cairo City household dust. Depending on the study’s aims and to guarantee representative sampling, 38 major districts in Cairo City are represented with at least 10 subsamples from each. Table 2 summarizes the descriptive statistical parameters (minimum, maximum, mean, and standard deviation) of the dry weight PTE concentrations in the analyzed indoor household dust samples. Generally, the mean concentrations of these PTEs were ranked in the declining sequence Fe (20,818 ppm) > Al (9092 ppm) > Mn (425 ppm) > Zn (419 ppm) > Cu (116.6 ppm) > Pb (99.3 ppm) > Cr (48.6 ppm) > V (45.7 ppm) > Ni (30.1 ppm) > Co (9.0 ppm) > As (4.0 ppm) > Mo (2.5 ppm) > Cd (1.0 ppm) > Hg (0.30 ppm).

Because there are no PTE guidelines for indoor dusts, our results were compared with the UCC element concentrations [51]. The mean concentrations of As, Cd, Cr, Cu, Hg, Mo, Ni, Pb, and Zn were higher than those of UCC [51], indicating that their sources were affected by anthropogenic activities. CV % indicates the relative variability of element levels in environmental samples. CV of 20% indicates low variability, CV of 20:50% indicates moderate variability, and CV of 50:100% indicates high variability [61,62]. The CV(%) values of the measured PTEs ranged from 23.9% to 148.1% (Table 2). An interesting point in Table 2 is that Hg exhibited the highest CV value (148.1%), indicating extremely high variability through sampling locations. Cd, Cu, and Pb exhibited relatively higher CV values (51.7, 50.3 and 52.2%, respectively) indicating possible pollution. On the other hand, Al, As, Co, Cr, Fe, Mn, Mo, Ni, V, and Zn showed moderate variability.

**Figure 2:**
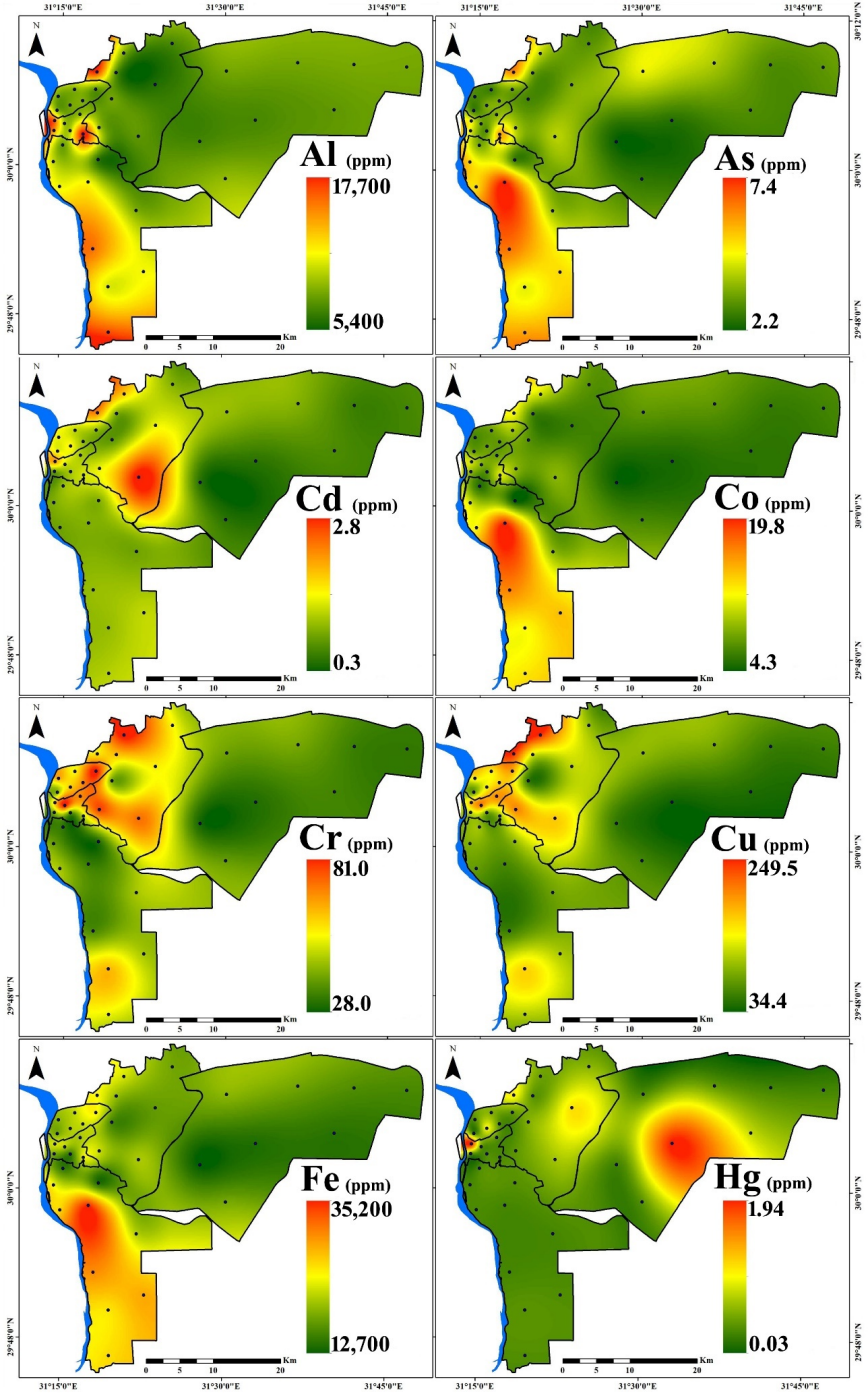

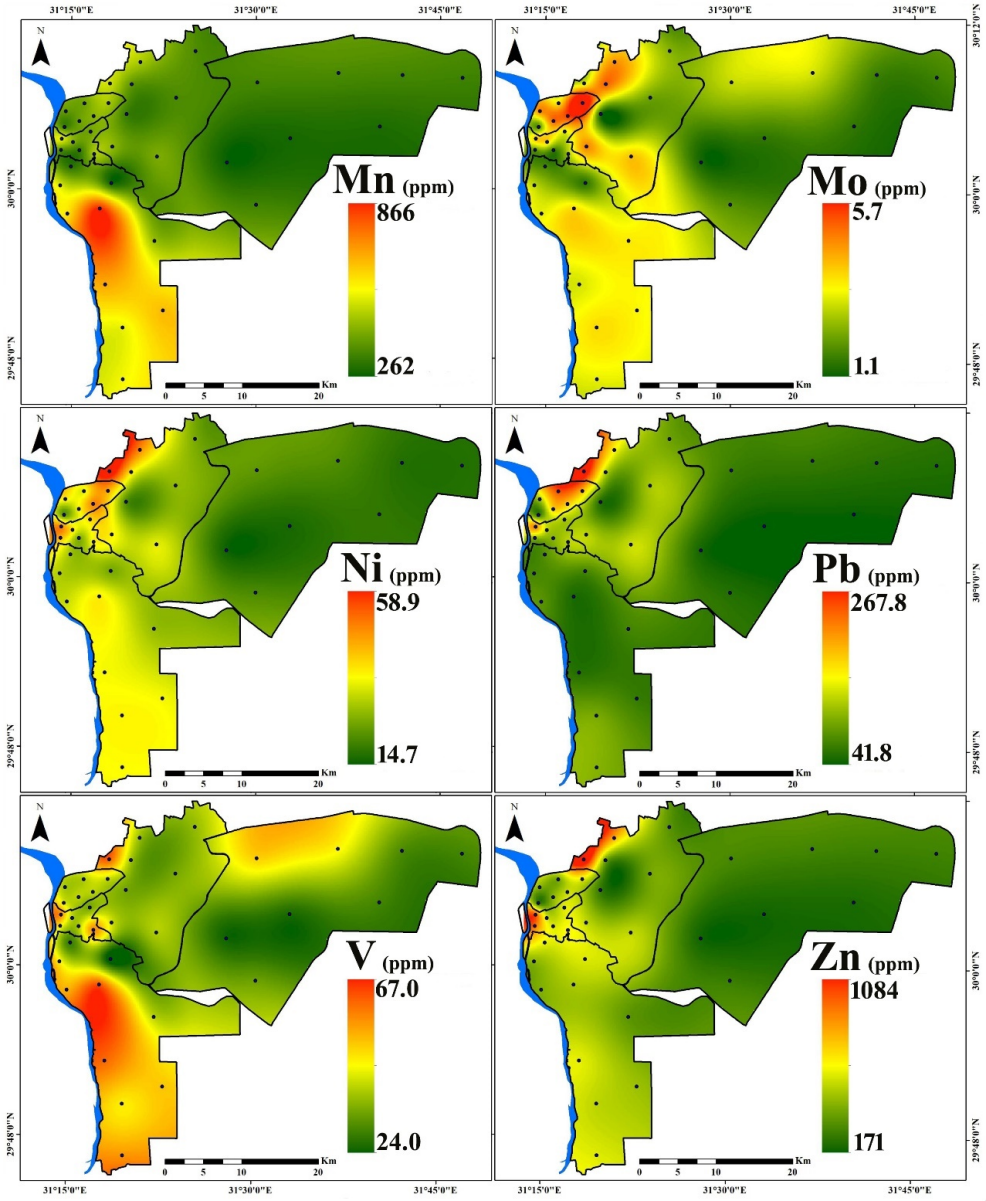
Spatial distribution of PTEs in household dust in Cairo City.

Figure 2 depicts the results of plotting PTE concentrations on spatial distribution maps. Elevated levels (hot spots) of Cd, Cr, Cu, Mo, Ni, Pb, and Zn concentrations were found mostly around eastern, northern, and western regions, which are characterized by higher traffic density, population density, and older buildings. On the other hand, high levels (hot spots) of As, Co, Fe, Mn, and V were mostly concentrated in the southern region, which is characterized by intense industrial activity. A high concentration of Hg was distributed in new Cairo and the northern region.

To put the levels of PTEs in Cairo’s indoor dust into perspective, they were compared with the levels of the same elements in indoor dust worldwide (Table 3). Table 3 shows that the mean PTE concentrations in our indoor dust samples were both higher and lower than those worldwide. For instance, Al mean concentration was greater than those reported in Slovenia (Maribor) [63], Greece (Athens) [64], and USA (Texas) [7]. As was higher than those reported in Nigeria (Lagos) [22], Nepal [65], and USA (Texas) [7]. Cd was higher than those reported in Alexandria and Kafr El-Sheikh [45], Saudi Arabia (Riyadh) [1], Qatar (Doha) [66], Nigeria (Lagos) [22], Turkey (Istanbul) [67], Iran (Ahvaz) [60], and Greece (Athens) [64]. Cu was higher than those reported in Kafr El-Sheikh [45], Saudi Arabia (Riyadh) [1], Iraq (Al-Fallujah) [68], Nigeria (Lagos) [22], Iran (Ahvaz) [60], and USA (Texas) [7]. Fe was lower than Qatar (Doha) [66], Nigeria (Lagos) [22], and Canada (Alberta) [69]. Mn was far lower than China (Huize) [12] and Nepal [65]. Ni was higher than those reported in Alexandria and Kafr El-Sheikh [45], Saudi Arabia (Riyadh) [1], Nigeria (Lagos) [22], Iran (Ahvaz) [60], Greece (Athens) [64], USA (Texas) [7], and Australia (Sydney) [70]. Pb was lower than Egypt (Alexandria) [45], Saudi Arabia (Jeddah) [14], Kuwait [71], Portugal (Estarreja) [25], China (Huize) [12], Nepal [65], Canada (Alberta) [69], and Australia (Sydney) [70]. Zn was higher than those reported in Egypt (Kafr El-Sheikh) [45], Saudi Arabia (Jeddah and Riyadh) [1,14], Iraq (Al-Fallujah) [68], Nigeria (Lagos) [22], Greece (Athens) [64], and USA (Texas) [7].

### 3.2. Contamination Levels

**Figure 3:**
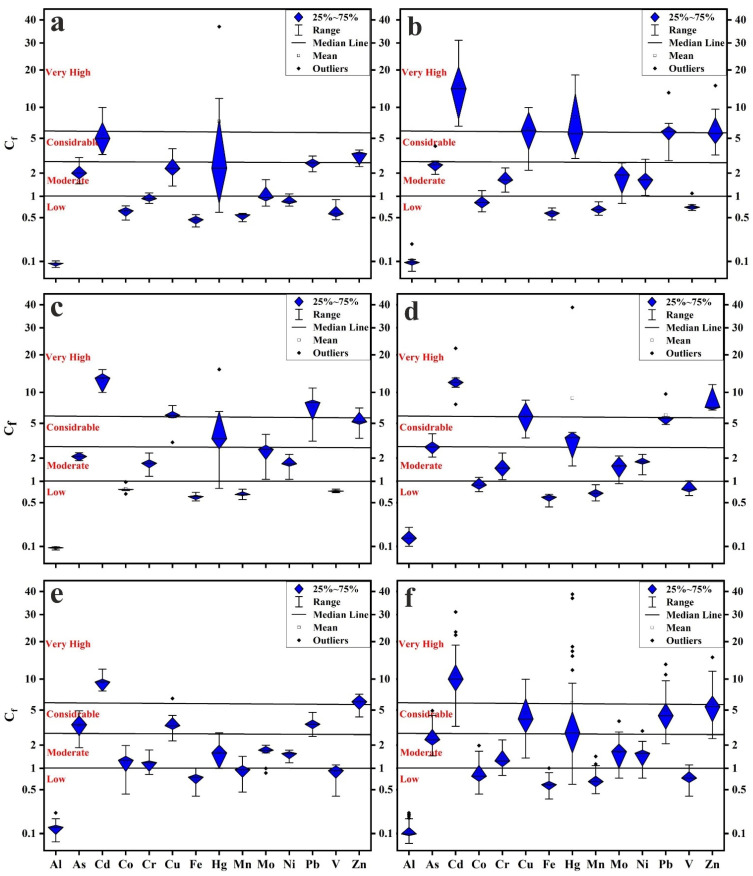
(**a**) Boxplots of *C_f_* values: New Cairo; (**b**) eastern region; (**c**) northern region; (**d**) western region; (**e**) southern region; (**f**) all samples.

The UCC element concentrations were used as the background values, and the *C_f_* and integrative *C_deg_* indices were applied to objectively analyze the contamination levels in the five administrative regions in Cairo City. The calculated *C_f_* values are presented in Table S2 and Figure 3. Altogether, the five regions were polluted to varying degrees by the measured PTEs. The lowest degrees of pollution were recorded for Al, Co, Fe, Mn, and V, while the highest degrees were recorded for Cd, Cu, Hg, Pb, and Zn, reaching considerably to very high pollution. Hg shows a wide range of *C_f_* values from low to very high pollution.

**Figure 4:**
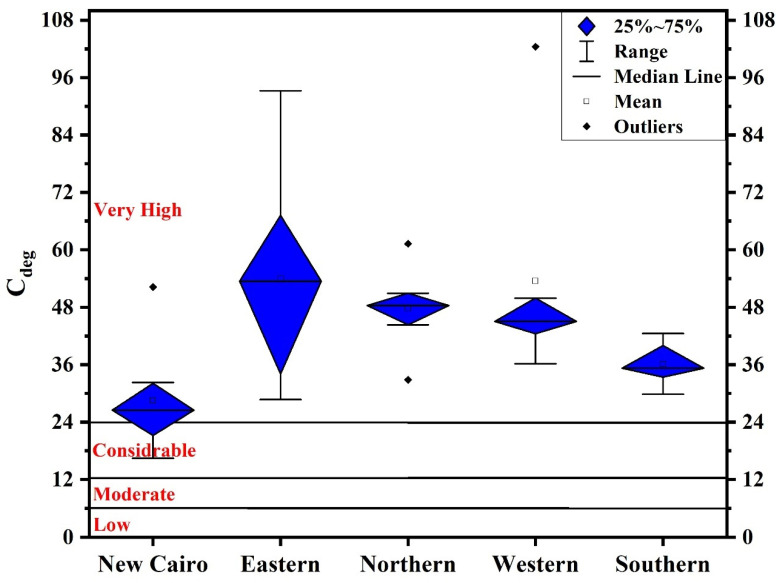
Boxplots of *C_deg_* values.

The calculated *C_f_*-based *C_deg_* values in the investigated five regions (Figure 4) indicate that New Cairo recorded the slightest degree of contamination, ranging from considerably to very high pollution. On the other hand, eastern, northern, western, and southern regions’ household dust were very highly polluted.

### 3.3. Correlations between PTEs

The multivariate statistical analysis including Pearson’s Correlation Coefficient matrix (PCC), Hierarchical Cluster Analysis (HCA) in Q mode, and Principal Component Analysis (PCA) were utilized to reveal and emphasize the correlation intensity and linkage between the analyzed PTEs.

Correlations values 0.00–0.19, 0.20–0.39, 0.40–0.59, 0.60–0.79, and 0.80–1.00 can be considered as very weak, weak, moderate, strong, and very strong correlations, respectively [73]. As shown in Table 4, very strong positive correlations were observed between Al–V (Pearson’s R = 0.81), As–Co (R = 0.91), As–Fe (R = 0.84), As–Mn (R = 0.86), As–V (R = 0.90), Co–Fe (R = 0.95), Co–Mn (R = 0.97), Co–V (R = 0.87), Cr–Cu (R = 0.88), Cu–Ni (R = 0.81), Fe–Mn (R = 0.97), Fe–V (R = 0.83), Mn–V (R = 0.81), and Ni–Zn (R = 0.87). Strong positive correlations were observed between Al–As (R = 0.78), Al–Co (R = 0.67), Al–Zn (R = 0.63), Cd–Cu (R = 0.69), Cd–Ni (R = 0.62), Cd–Pb (R = 0.65), Cd–Zn (R = 0.61), Cr–Mo (R = 0.79), Cr–Ni (R = 0.67), Cr–Pb (R = 0.64), Cu–Mo (R = 0.66), Cu–Pb (R = 0.79), Cu–Zn (R = 0.68), Mo–Ni (R = 0.67), Ni–Pb (R = 0.79), and Pb–Zn (R = 0.67). The most significant finding that can be deduced from these positive linear relations is the role played by Al, Fe, and Mn as scavenging elements in the distribution of PTEs, especially As, Co, V, and Zn [48,74]. The strong to very strong positive correlation between the measured PTEs indicates their close distribution and association and may suggest a shared source. It appears to imply that household dust with more elevated levels of one toxic element additionally contain higher levels of other PTEs.

**Figure 5:**
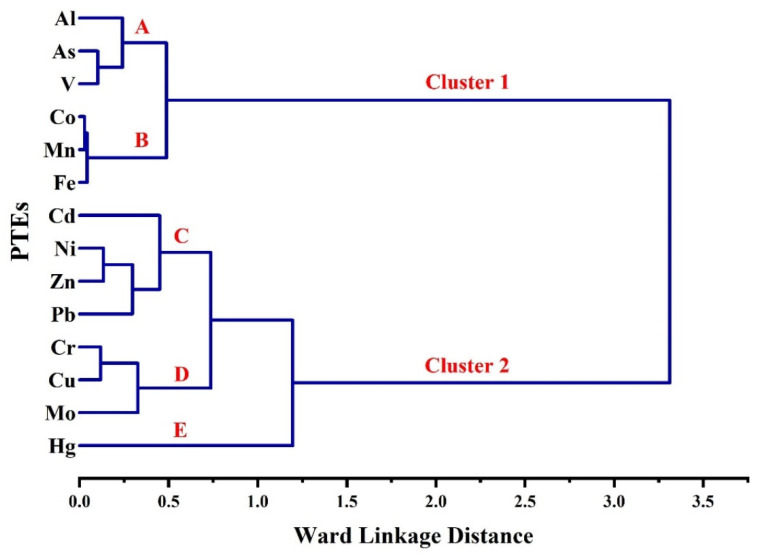
HCA dendrogram.

**Figure 6:**
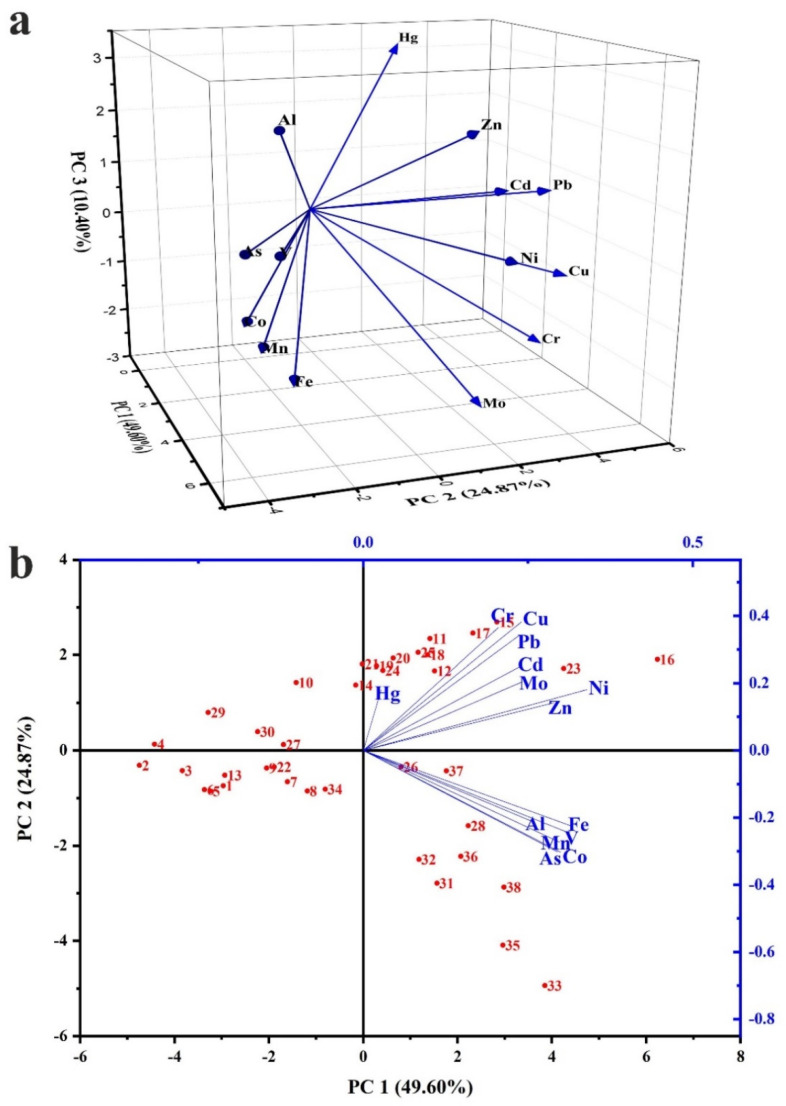
(**a**) PCA variable loading: 3D loading between the extracted 3 components; (**b**) 2D loading between PC1 and PC2 combined with sampling sites.

HCA (Figure 5) reduced data into two main clusters. Cluster (1) includes: (a) Al, As, and V and (b) Co, Mn, and Fe. Cluster (2) was subdivided into (c) Cd, Ni, Zn, and Pb; (d) Cr, Cu, and Mo; and (E) Hg. Figure 6 presents the PCA component. Three components, PC1 (49.60%; eigenvalue 6.44), PC2 (24.87%; eigenvalue 3.48), and PC3 (410.40%; eigenvalue 1.46), were extracted from PCA. The 3D plotting of the extracted three components positively confirms the association between Al, As, Co, Fe, Mn, and V (Figure 6a). The 2D plotting of PC1 and PC2 combined with sampling sites (Figure 6b) indicates that Al, As, Co, Fe, Mn, and V are more associated together in the southern region samples. It can be concluded that these elements originated from natural sources; this is in agreement with [22,75]. As enriched from intensive industrial activity in the southern region and adsorbed on Fe–Mn oxides surface [76].

PTEs in household dust can be attributed to indoor activities such as cooking, smoking, carpet, paper, clothing, cosmetic and personal care products, electric instruments, and cleaning products [65,71,77,78]. A substantial portion of the PTEs emitted by various outdoor activities can travel considerable distances via atmospheric particulate matter and enter the indoor environment in a variety of ways [71]. Al is geochemically stable, while Fe and Mn are geochemically related elements that are abundant in the earth’s crust and considered as major elements in soil minerals. The weathering of pre-existing rocks, sediments, and soils primarily releases these major elements [22,71,79] because the levels of Al, Fe, and Mn in the investigated household dust samples are not polluted and relatively deficient. These elements are probably of predominantly geogenic origin and were not enriched in the dust samples by anthropogenic activities. Some exceptions for Mn were recognized in some sites moderately polluted with Mn. Mn can be enriched by many anthropogenic sources such as Mn fungicides [80], Mn–Ni batteries [81], and pigment and paints [82]. Similarly, Co and V concentrations in the majority of the studied samples are below background levels and show a low degree of pollution, indicating that they originated from natural sources before being transported and settling in household dust.

Anthropogenic sources of As, Cd, Cr, Cu, Ni, Pb, and Zn include traffic emissions, braking engine wear, corrosion of vehicle parts, lubricating oils, coal, and fossil fuel combustion, building and construction materials, rubbers, pesticides, and industrial emissions [19,22,63,71,75,77,81,83]. Cr and Zn can be sourced from wood preservative furniture [12,65]. Chemical and pharmaceutical industries, coal combustion, municipal solid waste incineration, and cement manufacture are all anthropogenic sources of Hg. Building materials (interior decorations, paints, and fluorescent lamps), household appliances and electronic devices, LCD displays, monitors, batteries, clothes dryers, irons, washing machines, fluorescent bulbs, neon lights, and thermometers are other potential indoor sources [84].

### 3.4. Health Risk Assessment

**Figure 7:**
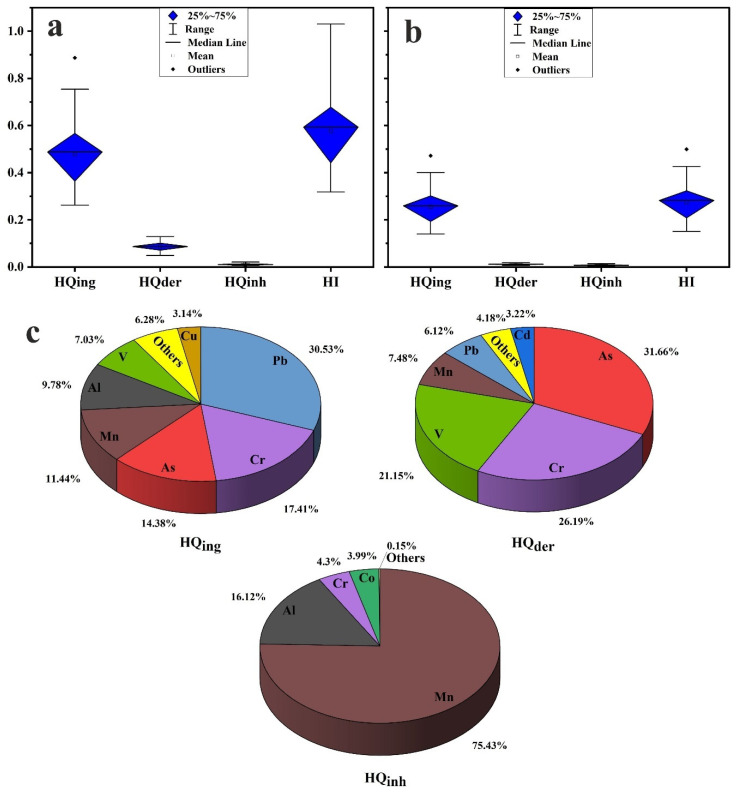
(**a**) Boxplots of noncancer risk for children; (**b**) for adults; (**c**) pie chart showing individual element contribution (%) for noncarcinogenic risk.

**Figure 8:**
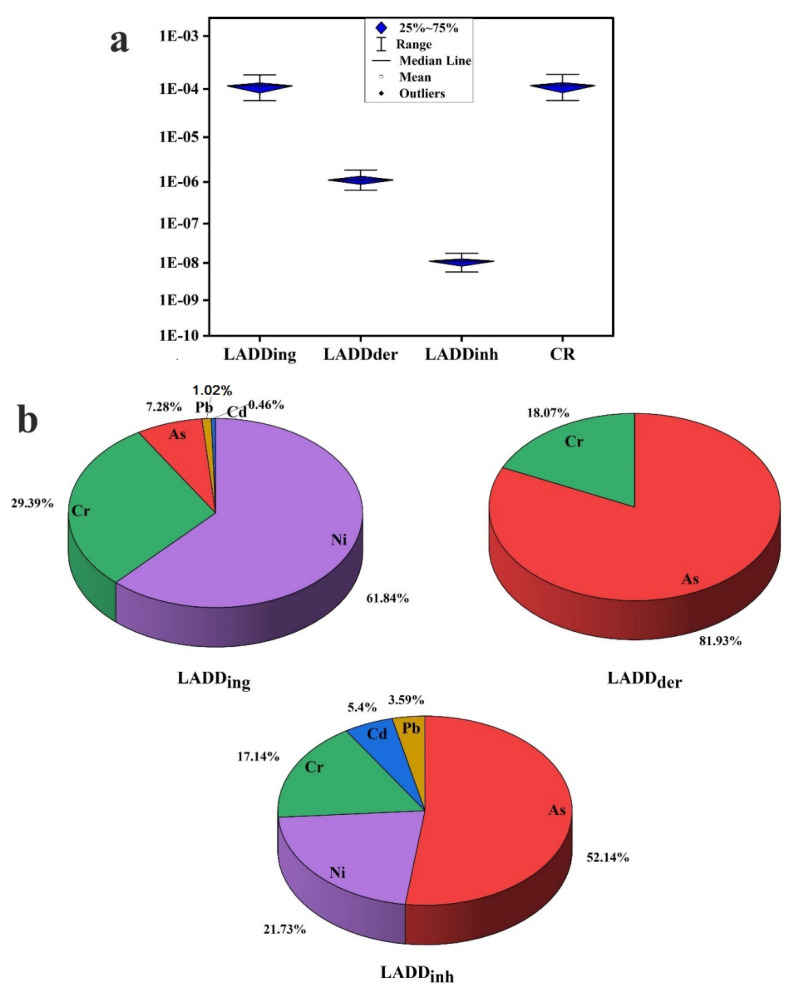
(**a**) Boxplots of cancer risk; (**b**) pie chart showing individual element contribution (%) for cancer risk.

Results of human health risk assessment show that the calculated *HQ_ing_*, *HQ_der_*, and *HQ_inh_* values for individual element (Table S3) and combined PTEs (Table 5; Figure 7a,b) in the household dust were less than one for children and adults. In addition, HI values for adults of the combined PTEs in the household dust were less than one, suggesting no potential noncancer risks (Table 5; Figure 7b). On the other hand, HI values for children were greater than those for adults; one site (site 16; Eastern region) recorded HI values higher than one, suggesting potential noncancer risks for children (Figure 7a). ⅀*HQ_ing_* was most likely to pose a noncancer risk of more than ⅀*HQ_inh_* and ⅀*HQ_der_*; this is consistent with several research findings [1,4,12,59,64,85]. The calculated individual element contribution (%) for children and adults noncarcinogenic risk revealed no differences in their contributions in the two age groups. As a result, we discuss them all together. Individual element contribution (%) for noncarcinogenic risk ⅀*HQ_ing_*, ⅀*HQ_der_*, and ⅀*HQ_inh_* is presented in Figure 7c. In’s ingestion route elements’ contribution is as follows: Pb (30.53%) > Cr (17.41%) > As (14.38%) > Mn (11.44%) > Al (9.78%) > V (7.03%) > Cu (3.14%) > Ni (1.61%) > Zn (1.50%) > Cd (1.07%) > Hg (1.07%) > Mo (0.54%) > Co (0.48) (Figure 8). For dermal contact route, elements’ contribution is as follows: As (31.66%) > Cr (26.19%) > V (21.15%) > Mn (7.48%) > Pb (7.12%) > Cd (3.22%) > Al (2.94%) > Hg (0.46%) > Cu (0.31%) > Zn (0.26%) > Ni (0.18%) > Mo (0.04%) > Co (0.02). For respiratory inhalation route, the contribution of Mn in ⅀*HQ_inh_* values was the largest, reaching 75.43%, followed by Al (16.12%), Cr (4.30%), and Co (3.99%).

In terms of carcinogenic risk, *LADD_ing_*, *LADD_der_*, and *LADD_inh_* values for individual elements were in the safe limit (Table S4). ⅀*LADD_ing_* values for As, Cd, Cr, Ni, and Pd were higher than 1 × 10^−4^ in the majority of the investigated sites, indicating a probability of cancer risk. On the other hand, ⅀*LADD_der_* values for As and Cr were higher than 1 × 10^−6,^ and ⅀*LADD_inh_* values for As, Cd, Cr, Ni, and Pd were lower than 1 × 10^−6^ (Figure 8a; Table 5). Alarmingly, CR values through the three routs of exposure were higher than 1 × 10^−4^ in the majority of the investigated sites, indicating a possible cancer risk to inhabitants in Cairo City. The CR risks via various exposure pathways were as follows: hand-to-mouth ingestion > dermal contact > respiratory inhalation. Individual element contribution (%) for carcinogenic risk ⅀*LADD_ing_*, ⅀*LADD_der_*, and ⅀*LADD_inh_* is presented in Figure 8b. The elements’ contributions are Ni (61.84%) > Cr (29.39%) > As (7.28%) > Pb (1.02%) > Cd (0.46%), As (81.93%) > Cr (18.07%), and As (52.14%) > Ni (21.73%) > Cr (17.14%) > Cd (5.40%) > Pb (3.59%) in ⅀*LADD_ing_*, ⅀*LADD_der_*, and ⅀*LADD_inh_*, respectively.

**Figure 9:**
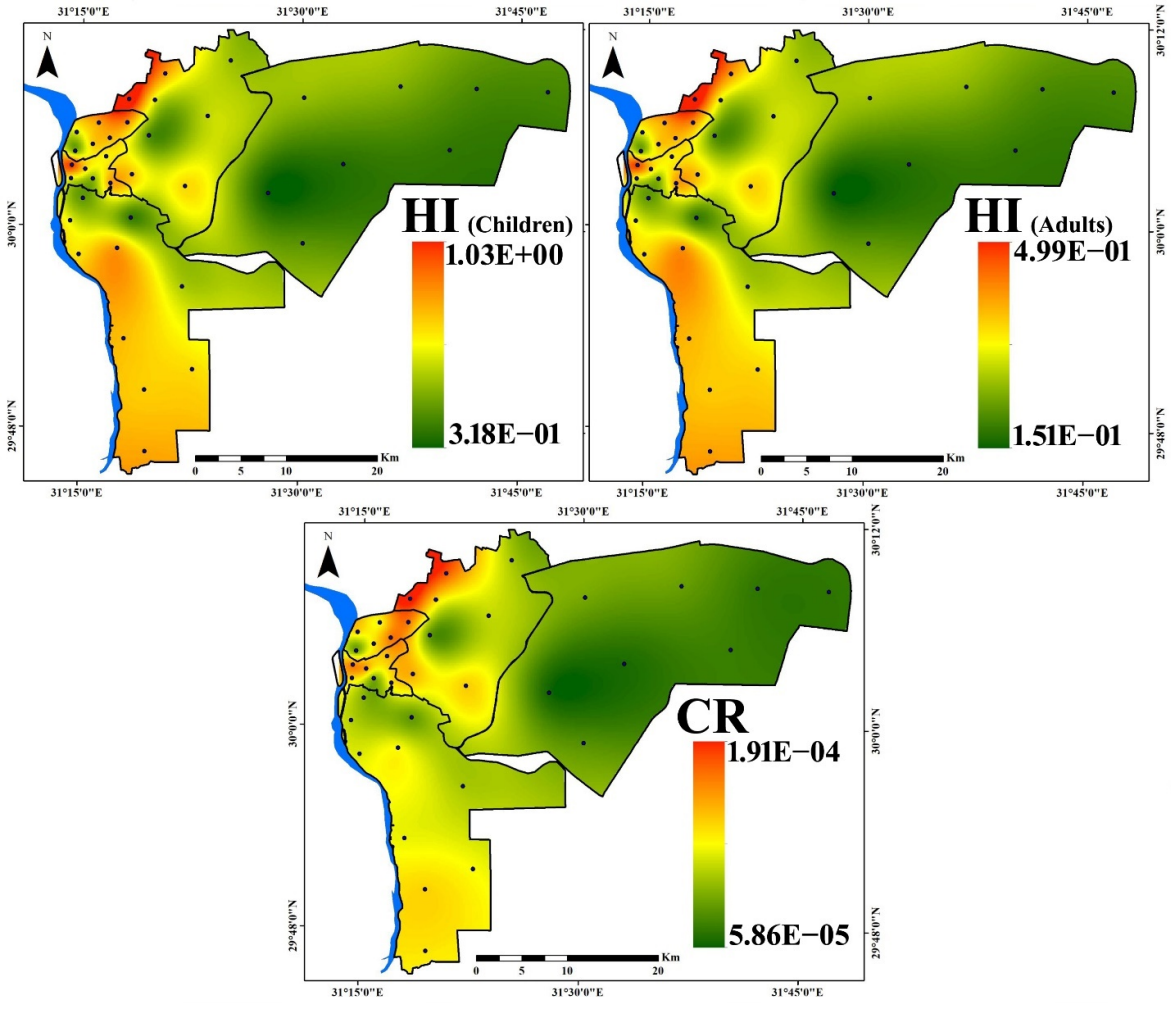
Spatial distribution of HI for children and adults and CR risks for the household dust exposure in Cairo City.

The spatial distribution maps of the calculated HI (children), HI (adults), and CR risks are presented (Figure 9) to inform decision makers about the riskiest districts so that mitigation measures could be implemented. The presented maps show the same distribution for noncancer and cancer risk, with hot spots concentrated in the eastern, northern, and western regions due to condensed road networks in these regions with permanent traffic congestion (Figure 1). In addition, the southern region showed considerable risk distribution due to the intensive industrial activity in this region. One of the most significant limitations of this investigation is the analysis of few composed samples and the undetermined indoor microenvironments. Additional investigation in highly polluted regions should include specific indoor microenvironments such as entrances, kitchens, living rooms, children’s rooms, and bedrooms to provide a more comprehensive analysis of household dust geochemistry in various microenvironments and to differentiate between PTE outdoor and indoor sources.

## 4. Conclusions

This study is the first one to comprehensively measure the chemical composition of household dust in Cairo City, Egypt. In general, the following important conclusions can be gained:

(1) The levels of As, Cd, Cr, Cu, Hg, Mo, Ni, Pb, and Zn surpassed the background values of UCC, indicating anthropogenic influences. The lowest degrees of pollution were recorded for Al, Co, Fe, Mn, and V, while the highest degrees were recorded for Cd, Cu, Hg, Pb, and Zn, reaching considerably to very high pollution.
(2) New Cairo recorded the slightest degree of contamination, ranging from considerably to very high pollution, while in other Cairo regions household dust is very high polluted. Elevated PTE concentrations in Cairo’s household dust may be due to industrial activities and heavy traffic emissions.
(3) The health risk assessment model revealed that the vital route of potential PTE exposure that leads to both noncarcinogenic and carcinogenic risks is ingestion, followed by dermal and inhalation pathways. The noncarcinogenic risk was generally in the safe range for adults’ exposure. Children are at risk in some sites, where HI values for the measured PTEs in household dust are higher than the recommended safe limit. Prolonged exposure to household dust in Cairo City would produce cancer risk to inhabitants.
(4) The critical contributors to noncancer risk are Pb, As, Cr, Mn, V, and Al. The main causes of cancer risk are Ni, As, and Cr.
(5) The study’s findings call for regular detection and assessment of the PTE concentrations and health risk in indoor dust in Cairo City, as well as initiation and facilitation of public health policy development, prevention of anthropogenic source pollutants, and implementation of specific control measures.

## Figures and Tables

**Table 1:** Definitions and reference values of human health risk model.

Term	Definition	Value	Ref.
*C_s_*	PTE concentration	Site specific	[52,53,54,55,56,57]
*IngR*	Dust ingestion rate (mg day^−1^)	200 (Children); 100 (Adults)	
*InhR*	Dust inhalation rate (m^3^ day^−1^)	7.6 (Children); 20 (Adults)	
*PEF*	Particle emission factor (m^3^ kg^−1^)	1.36 × 10^9^	
*SA*	Exposed skin area (cm^2^)	2699 (Children); 3950 (Adults)	
*SL*	Skin adherence factor (mg cm^2^ day^−1^)	0.2 (Children); 0.07 (Adults)	
*ABS*	Dermal absorption factor (year)	0.001 except for As (0.03)	
*ED*	Exposure duration (year)	6 (Children); 24 (Adults)	
*EF*	Exposure frequency (day year^−1^)	350	
*BW*	Average body weight (kg)	18.6 (Children); 70 (Adults)	
*AT*	Average life span for heavy metals (day)	noncarcinogens = ED×365; carcinogens = 70 × 365	
*CF*	Transformation factor	1 × 10^−6^	
*RfD_ing_*	Ingestion reference dose (mg kg day^−1^)	Al (1.00), As (3.00 × 10^−4^, Cd (1.00 × 10^−3^), Co (2.00 × 10^−2^), Cr (3.00 × 10^−3^), Cu (4.00 × 10^−2^), Hg (3.00 × 10^−4^), Mn (4.60 × 10^−2^), Mo (5.00 × 10^−3^), Ni (2.00 × 10^−2^), Pb (3.50 × 10^−3^), V (7.00 × 10^−3^), Zn (3.00 × 10^−1^)	[57,58,59]
*RfD_inh_*	Inhalation reference dose (mg m^3 −1^)	Al (1.43 × 10^−3^), As (3.00 × 10^−4^), Cd (1.00 × 10^−3^), Co (5.71 × 10^−6^), Cr (2.86 × 10^−5^), Cu (4.02 × 10^−2^), Hg (8.75 × 10^−5^), Mn (1.43 × 10^−5^), Ni (2.06 × 10^−2^), Pb (3.25 × 10^−3^), V (7.00 × 10^−3^), Zn (3.00 × 10^−1^)	[53,54,55,56,57,59,60]
*RfD_der_*	Dermal reference dose (mg kg day^−1^)	Al (1.00 × 10^−1^), As (1.23 × 10^−4^), Cd (1.00 × 10^−5^), Co (1.60 2), Cr (6.00 × 10^−5^), Cu (1.20 × 10^−2^), Hg (2.10 × 10^−5^), Mn (1.84 × 10^−3^), Mo (1.90 × 10^−3^), Ni (5.40 × 10^−3^), Pb (5.25 × 10^−4^), V (7.00 × 10^−5^), Zn (6.00 × 10^−2^)	[53,54,55,56,57,59,60]
*SLF_ing_*	Ingestion cancer slope factor (mg kg day^−1^)	As (1.5), Cd (0.38), Cr (0.5), Ni (1.7), Pb (0.0085)	[4,11,58]
*SLF_inh_*	Inhalation cancer slope factor (mg m^3 −1^)	As (15.1), Cd (6.3), Cr (0.42), Ni (0.84), Pb (0.042)	[4,57,60]
*SLF_der_*	Dermal contact cancer slope factor (mg kg day^−1^)	As (3.66), Cr (2)	[4,12]

**Table 2:** Descriptive statistics of PTEs (ppm) in household dust in Cairo City.

Region		Al	As	Cd	Co	Cr	Cu	Fe	Hg	Mn	Mo	Ni	Pb	V	Zn
New Cairo (*n* = 8)	Min.	6300	2.2	0.3	4.6	28.0	34.4	12,700	0.03	262	1.1	14.7	41.8	28.0	171
	Max.	8200	4.6	0.9	7.4	39.0	96.8	19,400	1.85	347	2.5	21.6	64.1	54.0	266
	Mean	7250	3.2	0.5	6.1	33.4	60.9	16,200	0.37	313	1.6	17.7	53.0	37.4	223
	St.D.	644	0.8	0.2	1.2	4.17	21.4	2389	0.63	34	0.5	2.5	7.9	9.5	40
Eastern (*n* = 8)	Min.	5400	2.9	0.6	6.1	40.0	54.5	16,200	0.15	326	1.2	20.6	56.6	38.0	234
	Max.	15,700	6.2	2.8	12.0	81.0	249.5	24,200	0.92	507	4.0	58.9	267.8	66.0	1084
	Mean	8413	3.9	1.4	8.5	60.1	155.7	20,175	0.41	402	2.7	34.2	127.1	44.8	486
	St.D.	3110	1.1	0.8	2.1	14.3	75.1	2796	0.31	64	1.1	12.7	62.6	9.0	279
Northern (*n* = 6)	Min.	7000	2.8	0.9	6.7	41.0	77.3	18,600	0.04	332	1.6	21.3	63.8	42.0	244
	Max.	7900	3.5	1.4	9.8	81.0	188.5	24,700	0.78	468	5.7	44.6	219.2	47.0	509
	Mean	7583	3.2	1.2	7.9	60.5	146.4	21,317	0.27	400	3.7	34.5	147.6	44.2	383
	St.D.	354	0.4	0.2	1.0	13.5	37.4	2187	0.27	46	1.4	8.1	54.0	1.7	93
Western (*n* = 6)	Min.	8100	3.1	0.7	7.2	37.0	86.9	15,100	0.08	319	1.4	24.6	97.3	38.0	485
	Max.	16,700	5.8	2.0	11.3	81.0	212.3	23,100	1.94	541	3.2	44.6	193.9	61.0	833
	Mean	11,683	4.2	1.2	9.2	55.2	149.4	20,233	0.45	418	2.4	35.9	121.9	49.3	605
	St.D.	3653	1.0	0.4	1.7	16.8	53.7	3020	0.73	79	0.7	6.7	36.3	9.8	161
Southern (*n* = 10)	Min.	5800	2.8	0.7	4.3	29.0	56.4	14,000	0.05	277	1.3	23.8	50.9	24.0	300
	Max.	17,700	7.4	1.1	19.8	61.0	164.3	35,200	0.14	866	3.0	34.7	94.9	67.0	515
	Mean	10,460	5.2	0.9	12.2	40.3	92.3	25,080	0.08	554	2.5	30.4	71.6	52.0	430
	St.D.	3434	1.5	0.2	4.8	9.5	30.4	6594	0.03	176	0.6	3.79	14.1	14.6	75
All Samples (*n* = 38)	Min.	5400	2.2	0.3	4.3	28.0	34.4	12,700	0.03	262	1.1	14.7	41.8	24.0	171
	Max.	17,700	7.4	2.8	19.8	81.0	249.5	35,200	1.94	866	5.7	58.9	267.8	67.0	1084
	Mean	9092	4.0	1.0	9.0	48.6	116.6	20,818	0.30	425	2.5	30.1	99.3	45.7	419
	St.D.	3065	1.3	0.5	3.4	15.9	58.6	4972	0.44	131	1.1	9.8	51.7	11.2	190
	CV (%)	33.7	32.3	51.7	38.3	32.8	50.3	23.9	148.1	31	41.6	32.5	52.1	24.6	45.4
UCC [51]		80,400	1.5	0.09	10	35	25	35,000	0.05	600	1.5	20	20	60	71

**Table 3:** Comparison between PTE concentrations in the household dust in Cairo City with those for indoor dust in other cities worldwide.

Location	*n*	Al	As	Cd	Co	Cr	Cu	Fe	Hg	Mn	Mo	Ni	Pb	V	Zn	Ref.
Egypt (Cairo)	*n* = 38	9092	4.0	1.0	9.0	48.6	116.6	20,818	0.3	425	2.5	30.1	99.3	45.7	419	This study
Egypt (Alexandria)	*n* = 5	NA	NA	0.8	3.2	29.2	141.0	NA	NA	237	NA	25.1	260.0	NA	771	[45]
Egypt (Kafr El-Sheikh)	*n* = 4	NA	NA	0.3	8.6	33.4	46.1	NA	NA	438	NA	23.2	24.8	NA	257	[45]
Saudi Arabia (Jeddah)	*n* = 10	NA	8.0	2.1	87.9	40.2	NA	8752	NA	392	NA	35.7	121.2	NA	343	[14]
Saudi Arabia (Riyadh)	*n* = 18	NA	NA	0.1	3.5	NA	59.2	6520	NA	434	NA	15.2	5.0	NA	94	[1]
Kuwait	*n* = 50	12,697	13.0	NA	12.5	90.0	209.0	14,453	NA	441	NA	56.0	158.0	NA	784	[71]
Qatar (Doha)	*n* = 12	19,812	7.2	0.7	12.3	91.8	192.9	20,504	NA	370	15.1	68.7	65.3	52.1	824	[66]
Iraq (Al-Fallujah)	*n* = 50	NA	NA	14.8	NA	289.5	65.0	NA	NA	NA	NA	105.7	75.6	NA	293	[68]
Nigeria (Lagos)	*n* = 40	32,000	3.3	0.5	NA	130.0	28.1	24,500	NA	368	NA	20.9	47.4	52.4	208	[22]
Turkey (Istanbul)	*n* = 31	NA	NA	0.8	5.0	55.0	156.0	NA	NA	136	NA	236.0	28.0	NA	832	[67]
Iran (Ahvaz)	*n* = 108	NA	NA	0.5	8.5	18.0	106.0	NA	NA	100	NA	12.0	74.0	NA	554	[60]
Japan	*n* = 100	15,700	NA	1.0	4.7	67.8	304.0	10,000	NA	226	2.1	59.6	57.9	24.7	920	[72]
Slovenia (Maribor) *	*n* = 27	7400	4.1	1.1	6.2	65.0	140.0	12,700	0.3	306	2.9	38.0	69.0	17.0	716	[63]
Portugal (Estarreja)	*n* = 19	10,500	11.1	1.0	5.5	70.6	261.0	11,900	0.4	178	3.2	67.0	174.0	15.0	1349	[25]
Greece (Athens)	*n* = 20	4217	4.0	0.5	NA	65.2	339.0	4913	0.4	128	NA	29.9	46.1	9.0	401	[64]
China (Huize)	*n* = 50	NA	88.5	25.2	NA	124.0	174.0	NA	1.9	1010	NA	NA	926.8	NA	3029	[12]
Nepal *	*n* = 24	NA	3.0	1.8	28.1	231.0	275.0	838	NA	1650	NA	122.0	233.0	NA	1260	[65]
USA (Texas)	*n* = 31	3738	3.6	1.9	NA	23.0	53.0	2939	NA	48	NA	12.0	38.0	NA	368	[7]
Canada (Windsor)	*n* = 60	11,453	8.1	3.0	NA	65.8	139.0	10,826	NA	171	2.7	50.5	65.0	14.9	677	[20]
Canada (Alberta)	*n* = 125	16,000	13.0	11.0	5.4	92.0	1900.0	26,000	NA	250	8.5	60.0	4500.0	15.0	14,000	[69]
Australia (Sydney)	*n* = 82	NA	NA	4.4	NA	83.6	147.0	5850	NA	76	NA	27.2	389.0	NA	657	[70]

*n* = Number of Samples; NA = Not Available; * Median.

**Table 4:** PCC matrix for PTEs in the investigated household dust (*n* = 38).

	Al	As	Cd	Co	Cr	Cu	Fe	Hg	Mn	Mo	Ni	Pb	V	Zn
Al	1.00	0.78	0.26	0.67	−0.03	0.10	0.56	0.06	0.57	0.03	0.51	0.27	0.81	0.63
As		1.00	0.28	0.91	−0.01	0.10	0.84	−0.09	0.86	0.22	0.50	0.11	0.90	0.49
Cd			1.00	0.24	0.59	0.69	0.27	0.30	0.22	0.42	0.62	0.65	0.35	0.61
Co				1.00	0.09	0.11	0.95	−0.13	0.97	0.33	0.49	0.09	0.87	0.40
Cr					1.00	0.88	0.27	0.07	0.14	0.79	0.67	0.64	0.11	0.39
Cu						1.00	0.25	0.13	0.14	0.66	0.81	0.79	0.16	0.68
Fe							1.00	−0.16	0.97	0.54	0.58	0.21	0.83	0.35
Hg								1.00	−0.10	−0.01	0.14	0.33	−0.03	0.22
Mn									1.00	0.42	0.51	0.12	0.81	0.35
Mo										1.00	0.67	0.56	0.32	0.30
Ni											1.00	0.79	0.55	0.87
Pb												1.00	0.27	0.69
V													1.00	0.48
Zn														1.00
	Very Weak			Weak			Moderate			Strong			Very Strong	

**Table 5:** Integrated noncancer and cancer risks values.

Noncancer Risk								
Children					Adults			
	**⅀*HQ_ing_***	**⅀*HQ_der_***	**⅀*HQ_inh_***	***HI***	**⅀*HQ_ing_***	**⅀*HQ_der_***	**⅀*HQ_inh_***	***HI***
Min	2.62 × 10^−1^	4.85 × 10^−2^	7.07 × 10^−3^	3.18 × 10^−1^	1.39 × 10^−1^	6.60 × 10^−3^	4.94 × 10^−3^	1.51 × 10^−1^
Max	8.87 × 10^−1^	1.28 × 10^−1^	2.09 × 10^−2^	1.03	4.72 × 10^−1^	1.75 × 10^−2^	1.46 × 10^−2^	4.99 × 10^−1^
Mean	4.79 × 10^−1^	8.60 × 10^−2^	1.14 × 10^−2^	5.77 × 10^−1^	2.55 × 10^−1^	1.17 × 10^−2^	7.95 × 10^−3^	2.74 × 10^−1^
**Cancer Risk**								
	**⅀*LADD_ing_***	**⅀*LADD_der_***	**⅀*LADD_inh_***	**CR**				
Min	5.80 × 10^−5^	6.35 × 10^−7^	5.72 × 10^−9^	5.86 × 10^−5^				
Max	1.89 × 10^−4^	1.87 × 10^−6^	1.78 × 10^−8^	1.91 × 10^−4^				
Mean	1.12 × 10^−4^	1.15 × 10^−6^	1.09 × 10^−8^	1.13 × 10^−4^				

## Data Availability

Not applicable.

## Supplementary Materials

The following supporting information can be downloaded at: https://www.mdpi.com/article/10.3390/toxics10080466/s1, Table S1: Samples distribution in administrative regions and districts in Cairo City.; Table S2: Calculated *C_f_* and *C_deg_* values; Table S3: Calculated noncancer *HQ_ing_*, *HQ_der_*, and *HQ_inh_* values. Table S4: Calculated cancer *LADD_ing_*, *LADD_der_*, and *LADD_inh_* values.
