# Supplementary material for: Spatial Distribution, Contamination Levels, and Health Risk Assessment of Potentially Toxic Elements in Household Dust in Cairo City, Egypt
Source: Toxics. 2022 Aug 11;10(8):466. doi: 10.3390/toxics10080466 (PMC9414935; doi:10.3390/toxics10080466)
Supplement: Supplementary file 1 [file toxics-10-00466-s001.zip › toxics-1860236-supplementary.pdf]

toxics MDPI

Supplementary Materials: Spatial Distribution, Contamination Levels and Health Risk Assessment of Potentially Toxic Elements in Household Dust in Cairo City, Egypt

Ahmed Gad, Ahmed Saleh, Hassan I. Farhat, Yehia H. Dawood and Sahar M. Abd El Bakey

Table S1. Samples distribution in administrative regions and districts in Cairo City.

Region.	Sample No.	Districts	Contributing Sources of Pollution
New Cairo (S = 82; *n* = 8)	1	New Cairo3 (S = 11)	Construction, Traffic emission, Residential
	2	New Cairo1 (S = 11)	
	3	New Cairo2 (S = 10)	
	4	New Cairo2 (S = 10)	
	5	Badr1 (S = 10)	
	6	Badr2 (S = 10)	
	7	Al-Shorouk1 (S = 10)	
	8	Al-Shorouk2 (S = 10)	
Eastern (S = 97; *n* = 8)	9	Al-Salam (S = 14)	Traffic emission, Residential
	10	Al-Nozha (S = 12)	
	11	Nasr City1 (S = 11)	
	12	Nasr City2 (S = 11)	
	13	Heliopolis (S = 11)	
	14	Ain Shams (S = 12)	
	15	Al-Marg (S = 13)	
	16	Al-Matariya (S = 13)	
Northern (S = 83; *n* = 6)	17	Al-Zaytoun + Al-Ameryia (S = 16)	Industrial Activity, Traffic emission, Residential
	18	Hadaeq Al-Qoba (S = 12)	
	19	Al-Zawya Al-Hamra (S = 14)	
	20	Al-Shrabiya (S = 13)	
	21	Al-Sahel (S = 13)	
	22	Rod Al-Farag + Shoubra (S = 15)	
Western (S = 86; *n* = 6)	23	Al-Azbakiya + Boulaq (S = 15)	Traffic emission, Residential
	24	Al-Waily (S = 13)	
	25	Bab Al-Shaariya + Al-Mosky (S = 16)	
	26	Abdin + Gharb Al-Qahira (S = 16)	
	27	Wasat Al-Qahira (S = 13)	
	28	Monshat Naser (S = 13)	
Southern (S = 125; *n* = 10)	29	Al-Mokattam (S = 11)	Industrial Activity, Traffic emission, Residential
	30	Al-Khalifa + Al-Sayida Zeinab (S = 15)	
	31	Misr Al-Qadima (S = 10)	
	32	Dar Al-Salam + Al-Maadi (S = 16)	
	33	Al-Basatin (S = 11)	
	34	Tora (S = 12)	
	35	Al-Masara (S = 12)	
	36	15th of May (S = 12)	
	37	Helwan (S = 13)	
	38	Al-Tebin (S = 13)	

S = subsamples; *n*= composite samples.

Table S2. Calculated C_f and C_{deg} values.

Sample No.	Region	C_f														C_{deg}
		**Al**	**As**	**Cd**	**Co**	**Cr**	**Cu**	**Fe**	**Hg**	**Mn**	**Mo**	**Ni**	**Pb**	**V**	**Zn**	
1	New Cairo	0.10	1.73	3.33	0.66	1.06	2.41	0.53	2.80	0.56	1.07	1.00	2.71	0.62	3.37	21.92
2		0.08	1.47	3.33	0.46	0.80	2.10	0.36	0.60	0.44	0.73	0.74	2.44	0.47	2.44	16.44
3		0.09	2.00	5.56	0.56	0.94	1.66	0.41	12.00	0.47	0.87	0.87	2.22	0.55	2.62	30.81
4		0.08	1.67	3.33	0.49	0.83	1.38	0.40	37.00	0.46	0.87	0.77	2.09	0.48	2.41	52.25
5		0.09	2.20	5.56	0.66	0.89	2.32	0.46	1.00	0.55	1.00	0.82	2.63	0.57	3.52	22.25
6		0.09	2.00	4.44	0.60	0.91	2.24	0.47	0.60	0.57	0.93	0.82	2.82	0.57	3.45	20.52
7		0.09	2.67	8.89	0.74	1.11	3.52	0.52	3.60	0.58	1.67	1.01	3.09	0.83	3.61	31.93
8		0.09	3.07	10.00	0.74	1.09	3.87	0.55	1.80	0.57	1.60	1.08	3.21	0.90	3.75	32.31
9	Eastern	0.10	1.93	6.67	0.76	1.31	2.81	0.51	3.00	0.58	1.00	1.13	4.19	0.73	4.04	28.76
10		0.07	2.33	14.44	0.61	1.54	4.72	0.50	18.40	0.58	1.20	1.28	5.45	0.63	4.75	56.51
11		0.09	2.73	31.11	0.88	2.00	6.74	0.59	9.20	0.68	2.13	1.58	6.09	0.72	6.38	70.92
12		0.11	2.73	14.44	0.89	2.14	8.20	0.63	3.20	0.73	2.47	1.73	5.91	0.77	6.46	50.41
13		0.09	1.93	6.67	0.72	1.14	2.18	0.46	6.40	0.54	0.80	1.03	2.83	0.63	3.30	28.73
14		0.09	2.33	8.89	0.66	1.71	5.25	0.57	3.40	0.63	2.67	1.78	5.87	0.70	4.87	39.42
15		0.10	2.80	18.89	1.09	2.31	9.98	0.65	4.80	0.78	2.40	2.23	7.12	0.68	9.66	63.48
16		0.20	4.13	23.33	1.20	1.57	9.92	0.69	17.00	0.85	1.67	2.95	13.39	1.10	15.27	93.26
17	Northern	0.09	2.27	10.00	0.98	2.31	7.54	0.71	4.00	0.78	3.80	2.06	8.53	0.75	7.17	50.97
18		0.10	2.33	10.00	0.75	1.91	6.63	0.64	2.80	0.72	2.87	2.23	8.17	0.73	6.49	46.37
19		0.10	1.87	12.22	0.78	1.51	5.99	0.63	6.60	0.66	1.93	1.59	10.96	0.72	4.90	50.46
20		0.09	2.33	15.56	0.78	1.66	5.74	0.60	15.60	0.65	2.67	1.66	8.01	0.73	5.23	61.30
21		0.09	1.93	14.44	0.67	1.80	6.15	0.55	2.60	0.64	2.47	1.74	5.44	0.70	5.15	44.37
22		0.10	1.87	14.44	0.77	1.17	3.09	0.53	0.80	0.55	1.07	1.07	3.19	0.78	3.44	32.87
23	Western	0.18	3.13	22.22	1.10	1.66	7.01	0.65	38.80	0.90	2.13	2.23	9.70	1.02	11.73	102.46
24		0.10	2.40	11.11	0.78	1.91	8.05	0.59	3.40	0.67	1.73	1.82	5.50	0.63	6.93	45.62
25		0.11	2.27	13.33	0.87	2.31	8.49	0.61	4.00	0.71	2.07	1.79	5.84	0.72	6.83	49.94
26		0.17	3.07	7.78	0.91	1.17	4.77	0.53	3.60	0.60	1.13	2.01	4.87	0.83	11.08	42.50
27		0.11	2.07	11.11	0.72	1.06	3.48	0.43	2.00	0.53	0.93	1.23	4.88	0.72	6.94	36.21
28		0.21	3.87	13.33	1.13	1.34	4.07	0.66	1.60	0.77	1.47	1.69	5.80	1.02	7.58	44.53
29	Southern	0.07	1.87	8.89	0.43	0.83	3.97	0.40	2.40	0.46	0.87	1.19	3.52	0.40	6.89	32.17
30		0.09	2.33	10.00	0.55	0.89	4.15	0.43	1.60	0.51	1.00	1.35	3.78	0.53	7.20	34.41
31		0.14	4.00	7.78	1.33	1.14	3.44	0.77	1.20	0.99	1.73	1.48	3.52	1.00	5.10	33.61
32		0.13	3.47	7.78	1.24	1.26	3.07	0.76	1.60	1.00	1.73	1.50	3.91	0.95	5.03	33.41
33		0.13	4.93	8.89	1.98	0.91	2.52	1.01	1.00	1.44	2.00	1.74	2.55	1.12	5.93	36.14
34		0.10	2.60	7.78	0.91	1.26	3.25	0.61	1.00	0.75	1.93	1.31	3.39	0.73	4.23	29.84
35		0.18	4.33	10.00	1.68	0.97	2.26	0.87	1.00	1.15	1.67	1.65	2.74	1.08	7.07	36.64
36		0.13	3.47	12.22	1.45	1.26	4.39	0.83	2.00	1.09	1.87	1.65	3.13	0.92	5.65	40.04
37		0.12	3.07	10.00	1.23	1.74	6.57	0.73	2.80	0.90	2.00	1.72	4.55	0.85	6.28	42.56
38		0.22	4.40	12.22	1.37	1.26	3.31	0.76	1.60	0.94	1.60	1.66	4.75	1.08	7.25	42.41

Table S3. Calculated non-cancer HQ_{ing}, HQ_{der}, and HQ_{inh} values.

	Children			Adults		
	**HQ_{ing}**	**HQ_{der}**	**HQ_{inh}**	**HQ_{ing}**	**HQ_{der}**	**HQ_{inh}**
**Al**	4.69×10^{-2}	2.53×10^{-3}	1.83×10^{-3}	2.49×10^{-2}	3.44×10^{-4}	1.28×10^{-3}
**As**	6.89×10^{-2}	2.72×10^{-2}	3.85×10^{-6}	3.66×10^{-2}	3.71×10^{-3}	2.69×10^{-6}
**Cd**	5.13×10^{-3}	2.77×10^{-3}	2.87×10^{-7}	2.73×10^{-3}	3.77×10^{-4}	2.00×10^{-7}
**Co**	2.32×10^{-3}	1.56×10^{-5}	4.53×10^{-4}	1.23×10^{-3}	2.13×10^{-6}	3.17×10^{-4}
**Cr**	8.34×10^{-2}	2.25×10^{-2}	4.89×10^{-4}	4.43×10^{-2}	3.07×10^{-3}	3.42×10^{-4}
**Cu**	1.50×10^{-2}	2.70×10^{-4}	8.36×10^{-7}	7.99×10^{-3}	3.68×10^{-5}	5.84×10^{-7}
**Hg**	5.13×10^{-3}	3.95×10^{-4}	9.83×10^{-7}	2.73×10^{-3}	5.38×10^{-5}	6.87×10^{-7}
**Mn**	5.48×10^{-2}	6.43×10^{-3}	8.57×10^{-3}	2.91×10^{-2}	8.76×10^{-4}	5.99×10^{-3}
**Mo**	2.59×10^{-3}	3.68×10^{-5}	-	1.38×10^{-3}	5.02×10^{-6}	-
**Ni**	7.75×10^{-3}	1.55×10^{-4}	4.20×10^{-7}	4.12×10^{-3}	2.11×10^{-5}	2.94×10^{-7}
**Pb**	1.46×10^{-1}	5.26×10^{-3}	8.80×10^{-6}	7.77×10^{-2}	7.16×10^{-4}	6.16×10^{-6}
**V**	3.37×10^{-2}	1.82×10^{-2}	1.88×10^{-6}	1.79×10^{-2}	2.47×10^{-3}	1.32×10^{-6}
**Zn**	7.19×10^{-3}	1.94×10^{-4}	4.02×10^{-7}	3.82×10^{-3}	2.64×10^{-5}	2.81×10^{-7}

Table S4. Calculated cancer $LADD_{ing}$, $LADD_{der}$, and $LADD_{inh}$ values.

	**$LADD_{ing}$**	**$LADD_{der}$**	**$LADD_{inh}$**
**As**	8.14×10^{-6}	9.39×10^{-7}	5.68×10^{-9}
**Cd**	5.12×10^{-7}	-	5.88×10^{-10}
**Cr**	3.29×10^{-5}	2.07×10^{-7}	1.87×10^{-9}
**Ni**	6.91×10^{-5}	-	2.37×10^{-9}
**Pb**	1.14×10^{-6}	-	3.91×10^{-10}
